# Detecting Community Structure by Using a Constrained Label Propagation Algorithm

**DOI:** 10.1371/journal.pone.0155320

**Published:** 2016-05-13

**Authors:** Jia Hou Chin, Kuru Ratnavelu

**Affiliations:** Institute of Mathematical Science, University of Malaya, Kuala Lumpur, Malaysia; King’s College London, UNITED KINGDOM

## Abstract

Community structure is considered one of the most interesting features in complex networks. Many real-world complex systems exhibit community structure, where individuals with similar properties form a community. The identification of communities in a network is important for understanding the structure of said network, in a specific perspective. Thus, community detection in complex networks gained immense interest over the last decade. A lot of community detection methods were proposed, and one of them is the label propagation algorithm (LPA). The simplicity and time efficiency of the LPA make it a popular community detection method. However, the LPA suffers from instability detection due to randomness that is induced in the algorithm. The focus of this paper is to improve the stability and accuracy of the LPA, while retaining its simplicity. Our proposed algorithm will first detect the main communities in a network by using the number of mutual neighbouring nodes. Subsequently, nodes are added into communities by using a constrained LPA. Those constraints are then gradually relaxed until all nodes are assigned into groups. In order to refine the quality of the detected communities, nodes in communities can be switched to another community or removed from their current communities at various stages of the algorithm. We evaluated our algorithm on three types of benchmark networks, namely the Lancichinetti-Fortunato-Radicchi (LFR), Relaxed Caveman (RC) and Girvan-Newman (GN) benchmarks. We also apply the present algorithm to some real-world networks of various sizes. The current results show some promising potential, of the proposed algorithm, in terms of detecting communities accurately. Furthermore, our constrained LPA has a robustness and stability that are significantly better than the simple LPA as it is able to yield deterministic results.

## Introduction

Network analysis emerges as a versatile tool that can be applied in various research fields such as sociology, bibliometric studies and transportation [1–3]. One of the most prominent properties in a network is its community structure. A community is defined as a group of nodes that are densely connected amongst themselves, while having lesser connection with the rest of the network [4]. Nodes that belong to a community indicate similar attributes among them. By studying the community structure of a network, one can get insight into the dominant features, attributes or functions of the nodes in the network. Hence, community detection has become a popular research topic over the decade. As a consequence, a large number of algorithms have been proposed and developed for the analysis of community structure in networks [5].

The label propagation algorithm (LPA) is a simple and fast community detection algorithm, that was originally proposed by Raghavan et al. [6]. The core idea of the LPA is to assign each node in a network into a community, to which most of its neighbouring nodes belong. Due to its simplicity, the LPA exhibits near linear complexity and it is a practical algorithm to detect communities in large networks with millions of nodes. Despite the simplicity and time efficiency of the LPA, it suffers from some instability detection due to its random update sequence and random tie breaking. Over the last decade, various modifications have therefore been implemented on the LPA in order to improve its stability and robustness. For example, Leung et al. [7] proposed hop attenuation and preferential linkage to break ties and prevent the trivial detection where all nodes are grouped in a single community. In addition, Barber and Clark [8] implemented modularity in the LPA and proposed a modified LPA that maximized modularity. Their method was improved upon by Liu and Murata [9], where they merged the communities to further maximize the modularity. Subsequently, Xie et al. [10] presented a LPA variant that can detect overlapping communities. The proposed algorithm is a speaker-listener based information propagation process (SLPA). In SLPA, a node can be either a speaker or a listener. As a listener, a node receives information from its neighbors. On the other hand, a node spreads the label with the highest frequency when it acts as a speaker. Apart from SLPA, Xie and Szymanski [11] also proposed a modified LPA called LabalRank in order to stabilize the LPA by incorporating a Marcov Cluster Algorithm into the LPA. Previously, they introduced an update rule that can speed up the LPA by avoiding unnecessary updates [12]. Here, they categorized the nodes in a network as being either passive or active. Passive nodes do not change label after update, while active nodes do change their label. Note that the algorithm stops when there is no active node. Zhang et al. [13] proposed a modified LPA with the capability for prediction of a percolation transition (LPAp). The effect of the prediction part in the LPAp will be to delay the formation of a monster size community. They also implemented an incomplete update condition in order to further reduce the computation time of the LPAp. A node influence based LPA (NIBLPA), which was proposed by Xing et al. [14], focuses in coping with the randomness of the LPA. In their work, they defined the node influence as a measurement for the importance of the nodes in a network. The node influence is used to decide the update sequence. Similarly, a label influence is defined to handle the random tie breaking dilemma in the LPA. In the recent work by Gaiteri et al. [15], they proposed a modified LPA called “SpeakEasy” to detect communities in biological networks effectively. Not only can “SpeakEasy” detect both overlapping and disjoint communities, it does not need any manual tuning of parameters. Furthermore, their algorithm is unique in the sense that it combines the usage of neighboring information (bottom-up approach) and the global information of a network (top-down approach). The combination of top-down and bottom-up approach will improve the accuracy of the detection. One of the latest modified LPA approach is the LINSIA by Wu et al. [16]. The main feature of the LINSIA is its ability to control the propagation process to uncover overlapping and hierarchical communities. This algorithm can also reveal the hubs and outliers in a network. Nonetheless, improvement on the stability and robustness of the LPA still remains an open question.

In this paper, we propose a few modifications to the LPA in order to tackle its instability while improving its accuracy. Initially, the proposed algorithm detects the main communities in a network by using the number of mutual neighbouring nodes. Nodes are now assigned into detected communities by using a constrained LPA. Those constraints are gradually relaxed until all the nodes are assigned into groups. At various stages of the algorithm, the quality of the detected communities is improved by switching nodes between communities or removing them from their current communities. Fixed update sequences, based on the degree of the nodes, are utilized to avoid the random label update problem. A parameter is also introduced to handle tie breaking during the propagation. Detected communities can also be merged in our approach if they fulfil certain conditions.

The performance of the proposed algorithm is compared to that for six other community detection methods: LPA, GANXiS, NIBLPA, Infomap, RN and Blondel. Here, LPA is the aforementioned simple label propagation algorithm of Raghavan et al. [6], while GANXiS (also known as SLPA) is a LPA variant by Xie et al. [10, 17, 18]. Although SLPA is known for detecting overlapping communities, GANXiS is capable of detecting both disjoint and overlapping communities in a network. NIBLPA [14], on the other hand, is a node influence based LPA variant. Infomap is a flow-based community detection method that optimizes the map equation [19]. The optimization of map equation requires the seeking of community structure in a network while minimizing the description length of a random walker. Ronhovde and Nussinov (RN) [20] proposed a method based on a multi-resolution spin-glass type Potts model, which is not affected by a resolution-limit. One of the most prominent features of the method is the ability to detect communities of heterogeneous sizes. Finally, Blondel et al. [21] devised a heuristic method that detects communities by optimizing the modularity of a network. That method is excellent in terms of it finding community structure with optimized modularity in a relatively short computational time.

We tested the present algorithm on three types of benchmark networks, namely the Lancichinetti-Fortunato-Radicchi (LFR) [22, 23], Relaxed Caveman (RC) [24, 25], and Girvan-Newman (GN) [4] benchmarks. We also implemented the algorithm to study some real-world networks of various sizes. We found that the present algorithm is capable of producing deterministic and accurate results. Furthermore, the results show that our variant of the LPA is substantially more robust and stable than the original LPA.

## Methods and Data

### Related Work

#### Label Propagation Algorithm

Given a network with n nodes, where node *x* with label at iteration *t* is denoted by *C*_*x*_(*t*), the LPA is described as following:

1. Initially, each node in a network has a unique label. Thus, *C*_*x*_(0) = *x*.
2. Set *t* = 1.
3. Obtain a list of updated labels for the nodes in a random order.
4. For each node in the list, let *C*_*x*_(*t*) = *f*(*C*_*x*_1__(*t*),…,*C*_*x*_*m*__(*t*),*C*_*x*_*m*+1__(*t*−1),…,*C*_*x*_*n*__(*t*−1)). *C*_*x*_1__(*t*),…,*C*_*x*_*m*__(*t*) are the labels of neighbouring nodes that have been updated in the current iteration while *C*_*x*_*m*+1__(*t* − 1),…,*C*_*x*_*n*__(*t* − 1) are the label of neighbouring nodes that are not yet updated. The function *f* returns the label which is adopted by most of its neighbouring nodes. Ties are broken randomly. This updating process is asynchronous. For a synchronous updating process, *C*_*x*_(*t*) = *f*(*C*_*x*_1__(*t* − 1),…,*C*_*x*_*m*__(*t* − 1)). Although the synchronous update is more time efficient than the asynchronous update, it encounters oscillations of the labels in the bipartite structure subgraph.
5. Terminate the algorithm if every node has the same label as most of its neighbouring nodes. Otherwise, set *t* = *t* + 1 and return to step (3).

### Algorithm

The proposed algorithm consists of 4 processes: mutual neighbour score and grouping, merging of detected communities, constrained LPA, and grouped nodes reallocation. Unlike the original LPA, for which the labels of all the nodes are updated in every iteration, we propose an update approach that handles the solo and grouped nodes separately. Solo nodes refer to nodes without any community, as contrasted to the grouped nodes. The function and details of these processes are explained in the following subsections.

#### Mutual neighbour score and grouping

**Fig 1:**
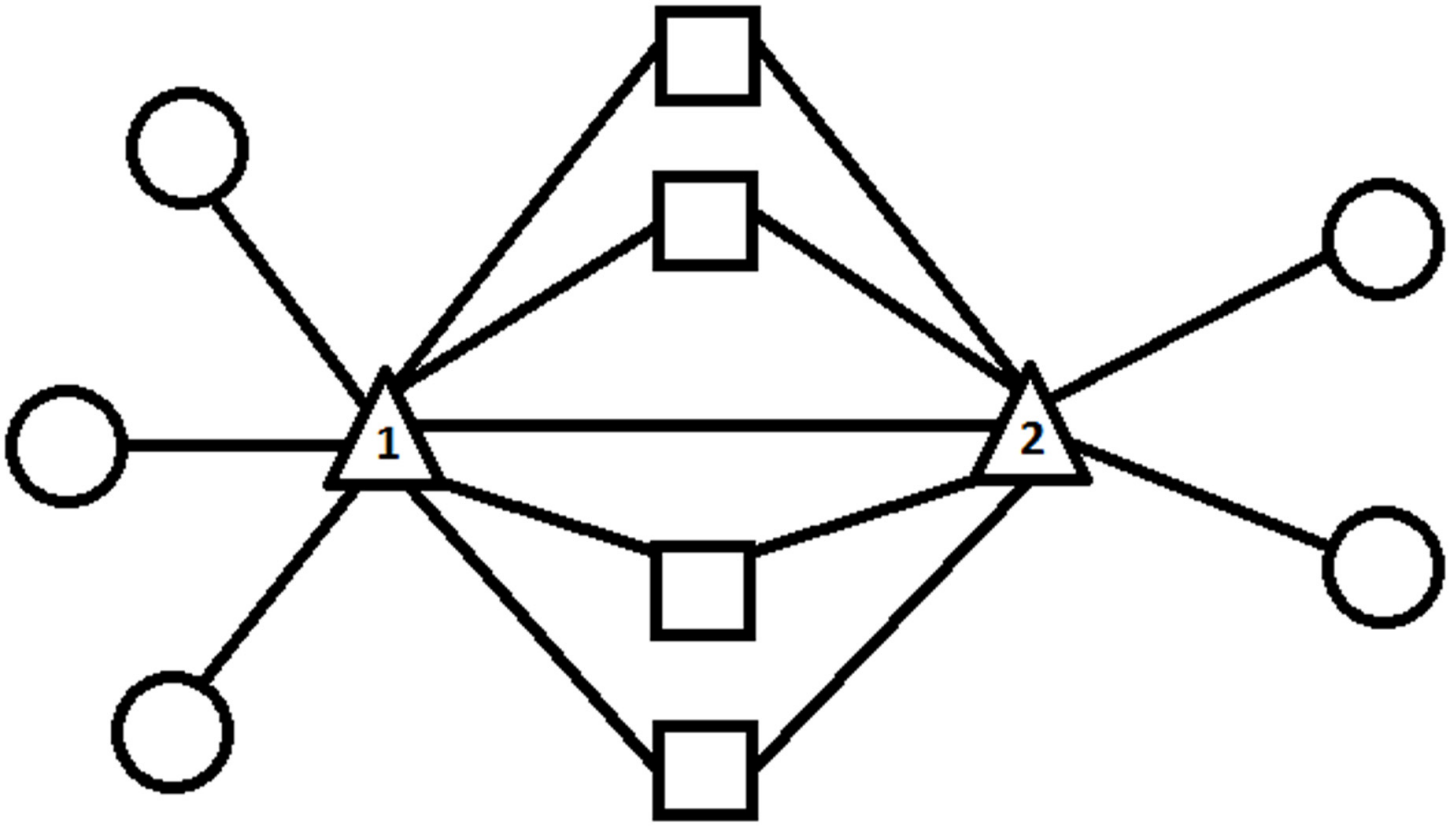
Example of a MNS calculation. Node 1 shares 4 neighbouring nodes (squares) with node 2. Since this is an undirected network, the MNS of node 1 to node 2 is 4, and vice versa.

Since a community is made out of densely connected nodes, a pair of nodes is likely to be in the same community if they share a large number of mutual neighbouring nodes. Based on this observation, a simple way of detecting the main communities in a network, by using the number of mutual neighbouring nodes, is utilised. The mutual neighbours score (MNS) of each node is calculated, where the MNS of a node is defined as the number of mutual neighbouring nodes it has with another node in a network. Fig 1 depicts the idea of the MNS.

The calculated MNS is used to form communities among the nodes in a network. This process consists of 2 parts. The first part is the highest to highest MNS grouping. Nodes that have the highest MNS with each other will be assigned into a group. In the second part, a node will simply adopt the label of a neighbour that shares the highest number of neighbours with it. In the case of a tie, where a node has similarly high MNS with multiple nodes, the labels of those nodes are observed. A node will adopt the label which is adopted by most of those nodes. Nodes are updated asynchronously, starting from the nodes with the lowest degree.

**Fig 2:**
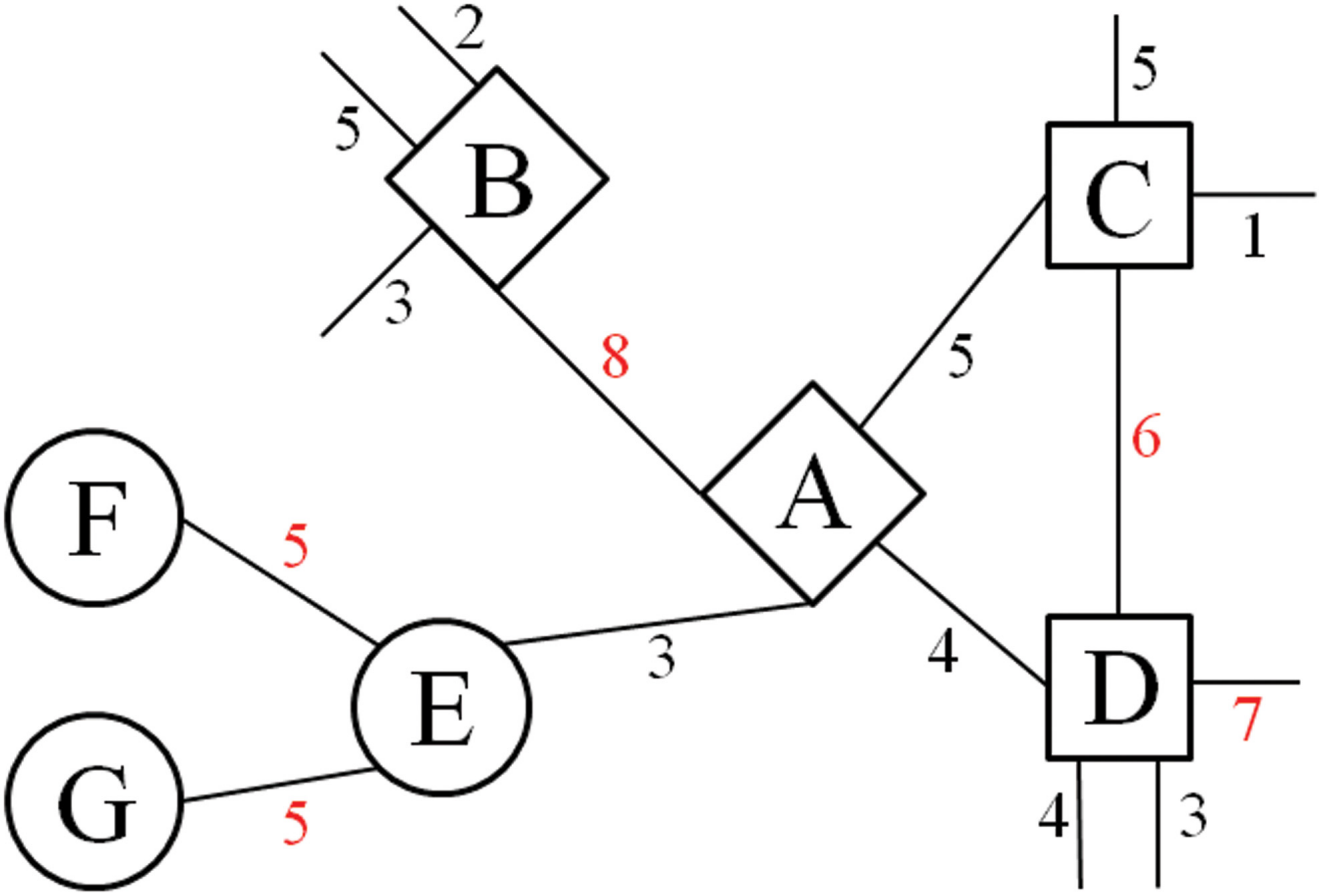
Example of a MNS grouping. Nodes A to G are divided into 3 groups (diamond, square and circle). The numbers attached to the edges are the MNS of a pair of nodes. Numbers in red indicates the highest MNS with another node. It must be noted that not all possible related nodes and edges are depicted in this figure.

Fig 2 shows a simple example describing the grouping of nodes based on the MNS. Node A has the highest MNS of 8 with node B, while node B also has the highest MNS of 8 with node A. Thus, nodes A and B will be assigned into a group. Node C has the highest MNS of 6 with node D, but the highest MNS of node D is 7. Consequently, node C adopts the label of node D. Node E has the highest MNS with multiple nodes (F and G). It happens that nodes F and G are in the same group. Hence, node E will adopt the label of nodes F and G.

#### Communities merging

Groups are merged if the following condition is satisfied:

$$\begin{array}{c} \frac{\text{inter}\;\text{community}\;\text{links}}{\text{no.}\;\text{of}\;\text{nodes}\;\text{in}\;\text{neighbouring}\;\text{community}}>\alpha \times \frac{\text{intra}\;\text{community}\;\text{links}}{\text{no.}\;\text{of}\;\text{nodes}\;\text{in}\;\text{target}\;\text{community}} \end{array}$$ (1)

For example, given two groups, A and B, with 5 and 7 nodes, respectively. Group A has 10 intra community links and 16 inter community links with group B. Since $\frac{16}{7}=2.286>\frac{10}{5}=2$, group A will be merged with group B. The *α* is a threshold parameter ranging from 0 to 1, which determines the difficulty of merging communities. As the value of *α* increases, the difficulty of merging communities also increases.

#### Constrained LPA (CLPA)

As mentioned earlier in this paper, the LPA suffers from instability that is caused by the randomness in the update sequencing, as well as the random tie breaking between multiple equally frequent labels. The most direct way of resolving the random update sequence is to use fixed update sequences. On the other hand, the solution for breaking a tie is the implementation of conditions for a node to decide the community that it belongs to. In this work, the capability of neighbouring nodes to accept new nodes into their communities is considered for addressing tie breaking. The capacity of a node to accept a neighbouring node into its community is defined as:

$$\begin{array}{c} \text{capacity}=\frac{\text{Number}\;\text{of}\;\text{inter}\;\text{community}\;\text{links}}{\text{Degree}} \end{array}$$ (2)

An example illustrating this concept is given in Table 1. Node J has 4 neighbouring nodes (K, L, M and N) that are distributed evenly among 2 communities, where K and L belong to C1 while M and N belong to C2.

**Table 1:** Example of the capacity of a given node for accepting a neighbouring node into its community.

Node	Inter Comm. Links	Degree	Capacity
K	5	10	0.500
L	9	16	0.563
M	5	8	0.625
N	4	12	0.333

The capability of C1 to accept node J into the community is the sum of the capacity of K and L, which is 1.063. On the other hand, C2 has the capacity of 0.958. Since C1 has higher capability in accepting new nodes, node J is assigned into C1.

In the original LPA, a node will adopt a label as long as that label is the most frequent label amongst the neighbouring nodes. However, it does not always increase or retain the density of communities. In fact, the density of a community may reduce drastically if it accepts nodes without restriction. In our approach, in order to maximize the density of a community, a node must fulfil certain conditions before it is assigned into a community. Those proposed independent conditions are as follows:

Condition 1: The number of links of a node to the nodes in a community is higher than the observed minimum number of intra community links in the community.
Condition 2: The number of links of a node to the nodes in a community is equal or higher than the observed minimum number of intra community links in the community.
Condition 3: The number of links of a node to the nodes in a community is higher than a threshold of observed minimum number of intra community links in the community. The threshold used in this work is half of the observed minimum number of intra community links in the community.
Condition 4: The number of links of a node to the nodes in a community is equal or higher than a threshold of observed minimum number of intra community links in the community.

It can be observed that the level of restriction is gradually relaxed in going from Conditions 1 to 4. Based on these conditions, a total of 7 asynchronous constrained LPA (CLPA) are designed to allocate solo nodes into detected communities. It must be noted that all the CLPA are executed recursively until there is a convergence in the label of all the nodes.

Constrained LPA 1

Starting from the smallest community, the labels of neighbouring solo nodes, for all the nodes in the community, are updated in a descending sequence, starting from the neighbouring node with the highest degree. A solo node can be allocated into a community if it fulfils Condition 1. The purpose of this CLPA, in prioritizing the small communities over the bigger ones, is to counterbalance the rapid growth of the big communities. The growth of the small communities here prevents them from being devoured by the big communities in later stages of the algorithm.

Constrained LPA 2

CLPA 2 is a less restrictive version of CLPA 1. A solo node can be allocated into a community if it fulfils Condition 2.

Constrained LPA 3

Starting from the solo node with the highest degree, the labels of nodes are updated in a descending sequence. A solo node is here allocated into the community, where most of its neighbouring nodes reside, provided that it fulfils Condition 3. If there is a tie between multiple communities, no action is taken by the solo node.

Constrained LPA 4

CLPA 4 is an extension of CLPA 3. In CLPA 4, a node that encounters a tie between multiple communities will choose the community with the higher capability for accepting a new node. Nonetheless, the node must still fulfil Condition 3 before it can be a member of the community.

Constrained LPA 5

Generally, CLPA 5 is similar to CLPA 1 and 2. In CLPA 5, a solo node is allocated into the community where most of its neighbouring nodes reside, provided it fulfils Condition 3. Furthermore, the modularity after a node enters a community must be higher than the modularity before it enters the community.

Constrained LPA 6

Starting with the grouped node with the highest number of neighbouring solo nodes, any neighbouring solo node that has MNS>0 with the grouped node is eligible to be in the same community as the grouped node. Nonetheless, it still needs to fulfil Condition 4 in order to be a member of the community.

Constrained LPA 7

CLPA 7 is a less restrictive version of CLPA 4. A solo node can here be allocated into a community as long as it fulfils Condition 4.

#### Grouped nodes reallocation (GNR)

GNR is implemented right after any CLPA for refining purposes. The function of GNR is the reallocation of a grouped node into another community or the removal a grouped node from its current community. The 4 conditions, which are introduced in CLPA, are also considered during the reallocation or removal of a grouped node.

Communities with only two nodes are unlikely to gain more nodes in the later stages of the algorithm. Furthermore, it may disrupt detection by introducing an undesirable tie situation. Hence, groups with only a couple of nodes are disbanded, where both grouped nodes become solo nodes. This process will be known as “dual removal” in this paper.

Grouped nodes reallocation 1

GNR 1 consists of two parts. In the first part, the number of intra community links of each node in a community is observed. A grouped node is removed from its current community if the number of intra community links of the node is less than the first quartile of the set of observed number of intra community links in a group. This process can only be executed once, as running it recursively will eventually remove all the nodes from a community.

In the second part, a process similar to CLPA 4 is carried out. In this case, the labels of the nodes are updated in an ascending sequence in terms of their degree. The labels of neighbouring nodes in a grouped node are observed, and one of these situations is encountered:

1. A grouped node has a label which a majority of its neighbouring nodes adopted.
2. A grouped node has a label which is not adopted by the majority of its neighbouring nodes.
3. There are multiple labels which are equally frequent among the neighbouring nodes.

In situation 1, the grouped node retains its current label. In situation 2, if a grouped node satisfies Condition 1, it will adopt the label to which most of its neighbouring nodes have. Otherwise, it is removed from its current community. In situation 3, if there are multiple communities sharing the similar highest capacity, the grouped node will be removed from its current community. If there is only one community with the highest capacity, a grouped node will switch to that community, provided it fulfils Condition 1. Otherwise, it is removed from its current community. All available situations 1 and 2 are addressed first, before handling situation 3. This process, together with the dual removal process, is executed recursively until convergence in the labels is achieved.

Grouped nodes reallocation 2

GNR 2 is basically GNR 1 without implementing the first part.

Grouped nodes reallocation 3

GNR 3 is exactly the same as GNR 2, except it implements Condition 3 instead of Condition 1.

Grouped nodes reallocation 4

GNR 4 is a reduced version of GNR 3, where there is no more node removal. Instead of removing a grouped node from its current community, it remains in its current community if it does not fulfil the relevant condition.

Grouped nodes reallocation 5

In this GNR, a grouped node that does not fulfil a condition will not be removed from its current community, and the dual removal process is also not implemented. In other words, communities with only two nodes are permitted to exist.

#### Constrained LPA with grouped nodes reallocation (CLPA-GNR)

**Fig 3:**
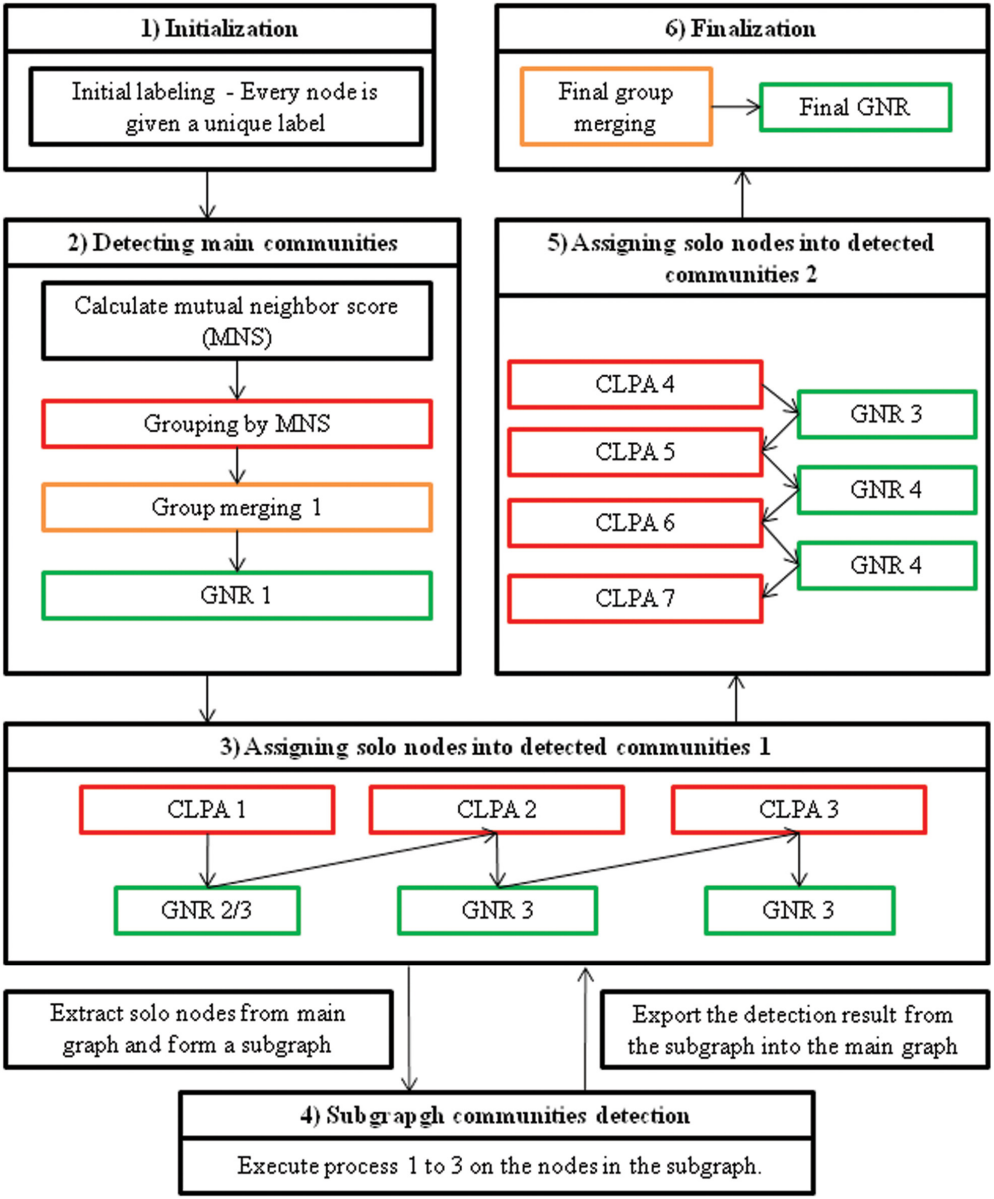
The flowchart of CLPA-GNR.

Our proposed algorithm (CLP-GNR) consists of 6 main parts, which in turn are a combination of the aforementioned processes. The flowchart of this algorithm is depicted in Fig 3. Part 1 is the initial labelling of all the nodes, where every node is given a unique label. In part 2, the MNS of each node is calculated. Concurrently, the MNS is used to detect initial communities. The detected communities are merged with the parameter *α* = 1. Afterwards, the grouped nodes undergo GNR 1. At the end of part 2, the main communities in a network are detected.

In part 3, solo nodes are allocated into the detected communities by using a series of CLPA (CLPA 1, CLPA 2 and CLPA 3). GNR 2 or GNR 3 is also implemented after each CLPA for refinement of the communities. If there is no solo node left at the end of part 3, the algorithm will execute part 6 directly, skipping parts 4 and 5.

If there exists at least 10 solo nodes after part 3, the remaining solo nodes and the corresponding edges are extracted from the main graph to form a subgraph as in part 4 of the process. Parts 1 to 3 are now executed on the subgraph and the result is exported into the main graph. After the main graph has imported the result from the subgraph, it will undergo all the processes in part 3 again. It must be noted that the GNR that is executed after CLPA 1 in part 3 is different pre- and post-part 4. GNR 2 is executed pre-part 4, while GNR 3 is executed post-part 4. The CLPAs in part 5 then allocate all the remaining solo nodes into the detected groups.

In part 6, detected communities are merged with the parameter *α* variying from 0.1 to 1 in descending order. Every time a group merging process with a particular *α* is executed, it is followed by the Final GNR. As the value of *α* decreases, more groups are merged and the number of final detected groups is consequently decreased. The group merging process continues until there is a drop in the modularity, which indicates that further merging of communities will not yield a better partition. The algorithm is then terminated.

### Time Complexity

As depicted in Fig 3 the CLPA-GNR consists of multiple processes. Each of the processes runs on a different time complexity. The time complexity of initialization for all nodes is *O*(*n*). Both the calculation of MNS and the detection of initial communities by using MNS have a time complexity of *O*(*m*). On the other hand, the time complexity of the merging of communities is *O*(*t*_*m*_ *m*), where *t*_*m*_ is the number of iterations that are needed for the convergence in the label of all the nodes. The final group merging process has a time complexity of *O*(*ut*_*m*_ *m*), as the parameter *α* is varying from 0.1 to 1. The term *u* is the number of times the group merging process is executed, before *α* reaches its optimal value.

The CLPA-GNR splits the original propagation process into two parts, which are the CLPA and GNR components. It is known that the time complexity of the original propagation process is *O*(*m*) [6]. By splitting the process into two parts, the time complexity of the process will also be divided. Hence, the time complexity of CLPA and GNR are *O*(*t*_*C*_ *m*_*C*_) and *O*(*t*_*G*_ *m*_*G*_), respectively. The terms *t*_*C*_ and *t*_*G*_ are similar to *t*_*m*_, while *m*_*C*_ and *m*_*G*_ refer to the number of edges that are involved in the CLPA and GNR processes. Furthermore, it should be noted that *m*_*C*_ ≤ *m*, *m*_*G*_ ≤ *m* and *m*_*C*_ + *m*_*G*_ = *m*. Even though the conditions (Conditions 1-4) are implemented in the CLPA and GNR processes, they do not affect the time complexity of the processes significantly.

The subgraph community detection process has the time complexity of the sum of the time complexity of the processes 1 to 3, as shown in Fig 3. However, in most of the cases, the size of the subgraph is relatively small (<10% of the main graph) so that the computation time for this process can be omitted. In conclusion, the total time complexity of the CLPA-GNR algorithm, excluding the subgraph communities detection process, is *O*(*n*)+*O*(*m*)+*O*(*t*_*m*_ *m*)+10*O(*t*_*c*_ *m*_*C*_)+10*O(*t*_*G*_ *m*_*G*_)+*O*(*ut*_*m*_ *m*)+*O*(*ut*_*G*_ *m*).

### Data Sets

We tested the proposed algorithm on 3 different synthetic networks, namely the Lancichinetti-Fortunato-Radicchi (LFR), Relaxed Caveman (RC) and Girvan Newman (GN) benchmarks. We also selected 9 real-world networks of various sizes as our test subjects. All the synthetic and real-world networks are undirected and unweighted, with disjoint ground truth communities.

#### Lancichinetti-Fortunato-Radicchi (LFR)

LFR benchmark networks are the most popular synthetic networks in the study of community detection. They feature heterogeneity in both the distribution of community sizes and the degree of the nodes. These properties are common in real-world networks, thus making LFR networks outstanding benchmarks. The mixing parameter, *μ*, refers to the average percentage of edges that link a couple of nodes from different communities. As the value of *μ* increases, the community structure of a network will weaken.

Two main groups of LFR networks with different sizes are generated. “Big networks” (B) consist of 5000 nodes while “small networks” (S) have 1000 nodes. Each main group has 4 subgroups with different community sizes and average degree. “Small communities” (SC) and “large communities” (LC) refer to networks with community sizes ranging from 10 to 50 and 20 to 100, respectively. On the other hand, “small average degree” (SK) and “large average degree” (LK) refer to networks with nodes of average degree of 10 and 20, respectively. Finally, each subgroup contains 8 networks with *μ* ranging from 0.1 to 0.8. In conclusion, a total of 64 LFR networks are generated. The parameters used when generating these networks are summarized in Table 2.

**Table 2:** Summary of the generated LFR networks.

Parameter	Definition	Values
N	Number of nodes	S = 1000; B = 5000
k	Average degree	SK = 10; LK = 20
maxk	Maximum degree	50
minc	Minimum community size	SC = 10; LC = 20
maxc	Maximum community size	SC = 50; LC = 100
*μ*	Mixing parameter	0.1–0.8
*γ*	Exponent for the degree sequence	2
*β*	Exponent for the community size distribution	1

#### Relaxed Caveman (RC)

The RC benchmark network consists of 512 nodes that are divided into 16 communities of highly heterogeneous sizes. Initially, a RC network has 16 isolated k-cliques. The community structure of the network is progressively weakened by removing a portion of edges from each clique and rewiring them to neighbouring cliques. The portion is determined by a parameter called Degradation (*D*) [26]. In this paper, the value *D* is varied from 10% to 80%.

#### Girvan Newman (GN)

The GN benchmark network consists of 128 nodes that are divided equally into 4 communities of 32 nodes each. Let *P*_*in*_ and *P*_*out*_ represent the probabilities of an edge being an intra community edge or inter community edge, that connects a couple of nodes from the same community or different communities. It is necessary to maintain the average degree of a node at around 16, when choosing the probabilities. GN benchmark networks can be generated by using a LFR benchmark networks generator as shown in Table 3. *P*_*in*_ and *P*_*out*_ are represented by *μ* when generating GN networks using a LFR generator. Low *μ* values indicate a high *P*_*in*_ and a low *P*_*out*_.

**Table 3:** Summary of generated GN networks.

Parameter	Definition	Values
N	Number of nodes	128
k	Average degree	16
maxk	Maximum degree	16
minc	Minimum community size	32
maxc	Maximum community size	32
*μ*	Mixing parameter	0.1–0.8
*γ*	Exponent for the degree sequence	0
*β*	Exponent for the community size distribution	0

#### Real-world networks

The real-world networks that are used in this work are summarized in Table 4. These networks are often employed in the testing of community detection methods.

**Table 4:** Summary of the real-world networks.

Network	Nodes	Edges	Ground Truth	Reference
Karate	34	78	Yes	[27]
Dolphins	62	159	Yes	[28]
Pol-books	105	441	Yes	[29]
Football	115	613	Yes	[29]
Jazz	198	2742	No	[30]
E. Coli	418	519	No	[31]
Email	1133	5451	No	[32]
Power	4941	6494	No	[24]
Collaboration	8361	15751	No	[33]

### Evaluation Criteria

In this work, we use the normalized variation of information (*NVI*) [34, 35], normalized mutual information (*NMI*) [36], modularity (*Q*) [29] and modularity density (*Q*_*ds*_) [37] as the criteria to evaluate the quality of the detected communities. Some of the real-world networks, as well as all the synthetic networks such as the LFR, RC and GN networks, have ground truth community structure. For networks with known community structure, *NVI* is used to measure the quality of our results for community detection on the networks. However, most of the real-world networks do not come with ground truth community structure. In those cases, we use *Q* and to evaluate the quality of the detected communities.

#### Variation of information

The variation of information (*VI*) is defined as:

$$\begin{array}{cc} VI(X,Y) & =H(X)+H(Y)-2I(X,Y)\\ & =H(X|Y)+H(Y|X) \end{array}$$ (3)

where *X* and *Y* are partitions of the same network. *H*(*X*) and *H*(*Y*) represent the entropy of *X* and *Y*, while *H*(*X*|*Y*) and *H*(*Y*|*X*) are the conditional entropies. *I*(*X*, *Y*) is the mutual information between *X* and *Y*. *VI*(*X*, *Y*) can also be written as:

$$\begin{array}{c} VI\left(X,Y\right)=-\sum_{x\in X,y\in Y}P\left(x,y\right)log\frac{P(x,y)}{P(y)}-\sum_{x\in X,y\in Y}P\left(x,y\right)log\frac{P(x,y)}{P(x)} \end{array}$$ (4)

where *P*(*x*) and *P*(*y*) are the probability of a node belonging to community *x* or *y* in partition *X* or *Y*, respectively. *P*(*x*, *y*) represents the probability of a node that belongs to community *x* and *y* in partition *X* and *Y*.

The maximum value of *VI* is *log n*. Hence, *VI* can be normalized by dividing it with *log n*, provided that the comparison is done on networks with a similar size. The value of the normalized *VI* (*NVI*) ranges from 0 to 1, where *NVI* is 0 if two partitions are identical.

#### Normalized Mutual Information

The normalized mutual information (*NMI*) is defined as:

$$\begin{array}{c} NMI\left(X|Y\right)=\frac{-2\sum_{i=1}^{C_{X}}\sum_{i=1}^{C_{X}}N_{ij}\log\frac{N_{ij}N}{N_{i.}N_{.j}}}{\sum_{i=1}^{C_{X}}N_{i.}\log\frac{N_{i.}}{N}+\sum_{j=1}^{C_{Y}}N_{.j}\log\frac{N_{.j}}{N}} \end{array}$$ (5)

where *C*_*X*_ and *C*_*Y*_ denote the number of communities in partition *X* and *Y*. The matrix *N* has rows and columns that correspond to communities in *X* and *Y*, respectively. The element of *N*, *N*_*ij*_ is the number of mutual nodes between communities *i* and *j*. The terms *N*_*i*._ and *N*_.*j*_ are the sum over rows and columns.

The value of *NMI* ranges from 0 to 1, where *NMI* = 1 when partition *X* is identical to partition *Y*. If partition *X* is total independent of partition *Y*, then *NMI* = 0.

#### Modularity

The modularity (*Q*) is defined by:

$$\begin{array}{c} Q=\frac{1}{2m}\sum_{i,j\in V}\left(A_{ij}-\frac{d_{i}d_{j}}{2m}\right)\times \delta \left(c_{i},c_{j}\right) \end{array}$$ (6)

where *m* represents the number of edges. Given a network with *n* nodes, *A*_*ij*_ is an *n* × *n* adjacency matrix, where *A*_*ij*_ = 1 if node *i* is linked to node *j*. Otherwise, *A*_*ij*_ = 0. *δ*(*c*_*i*_, *c*_*j*_) is a piecewise function that is defined as:

$$\begin{array}{c} \delta \left(c_{i},c_{j}\right)=\left\{\begin{array}{cc} 1 & c_{i}=c_{j}\\ 0 & c_{i}\neq c_{j} \end{array}\right. \end{array}$$ (7)

The value of *Q* ranges from 0 to 1, where networks with a strong community structure have a higher *Q* value.

#### Modularity Density

Modularity density (*Q*_*ds*_) is a modified modularity that resolves the resolution limit problem [38] as well as the tendency of splitting large communities into smaller communities. The *Q*_*ds*_ for undirected networks is defined as:

$$\begin{array}{c} Q_{ds}=\underset{c_{i}\in C}{\sum }\left[\frac{m_{c_{i}}^{in}}{m}d_{c_{i}}-{\left(\frac{2m_{c_{i}}^{in}+m_{c_{i}}^{out}}{2m}d_{c_{i}}\right)}^{2}-\sum_{\underset{c_{j}\neq c_{i}}{c_{j}\in C}}\frac{m_{c_{i},c_{j}}}{2m}d_{c_{i},c_{j}}\right] \end{array}$$ (8)

$$\begin{array}{c} d_{c_{i}}=\frac{2m_{c_{i}}^{in}}{n_{c_{i}}\left(n_{c_{i}}-1\right)} \end{array}$$ (9)

$$\begin{array}{c} d_{c_{i},c_{j}}=\frac{m_{c_{i},c_{j}}}{n_{c_{i}}n_{c_{j}}} \end{array}$$ (10)

where *m* is the total number of edges, $m_{c_{i}}^{in}$ is the number of intra community edges, $m_{c_{i}}^{in}$ is the number of inter community edges, and *m*_*c*_*i*_, *c*_*j*__ is the number of edges between communities *c*_*i*_ and *c*_*j*_. The terms, *n*_*c*_*i*__ and *n*_*c*_*j*__, refer to the number of nodes in communities *i* and *j*. *d*_*c*_*i*__ is the internal density of community *c*_*i*_, while *d*_*c*_*i*_, *c*_*j*__ is the pair-wise density between *c*_*i*_ and *c*_*j*_.

## Results and Discussion

In this section, the performance of the CLPA-GNR is compared with the other algorithms in both the synthetic and real-world networks. It must be noted that all of the reported detections are in the form of disjoint communities.

### Analysis on the Synthetic Networks

**Fig 4:**
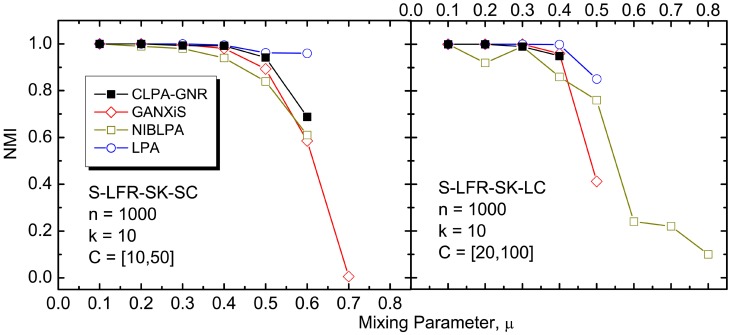
The *NMI* comparison on undirected and unweighted small LFR benchmark networks with small average degree. The size of the networks, size of the communities and the average degree are *n*, *C* and *k*, respectively.

**Fig 5:**
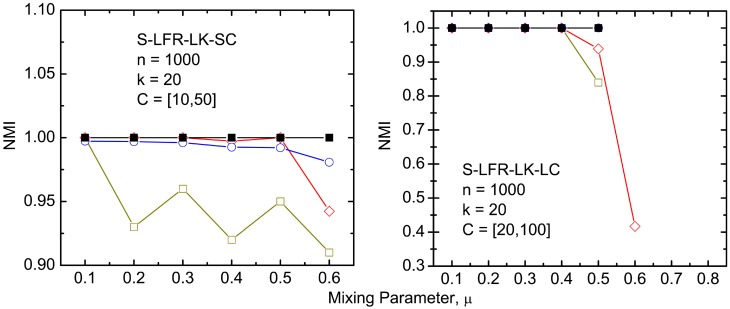
The *NMI* comparison on undirected and unweighted small LFR benchmark networks with large average degree. The legend is the same as in Fig 4.

**Fig 6:**
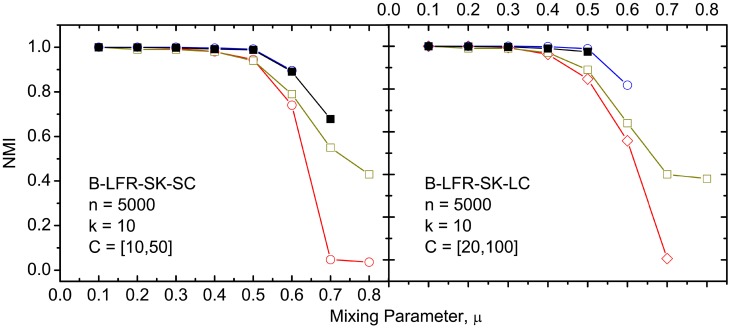
The *NMI* comparison on undirected and unweighted big LFR benchmark networks with small average degree. The legend is the same as in Fig 4.

**Fig 7:**
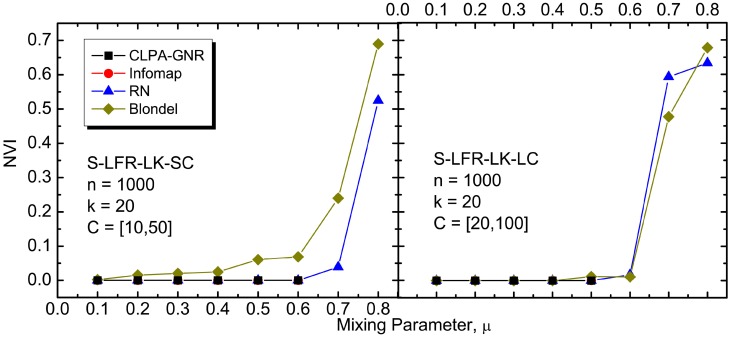
The *NVI* comparison on undirected and unweighted small LFR benchmark networks with large average degree.

**Fig 8:**
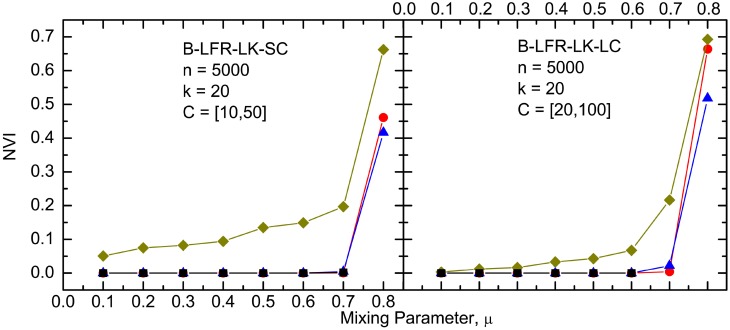
The *NVI* comparison on undirected and unweighted big LFR benchmark networks with large average degree. The legend is the same as in Fig 7.

**Fig 9:**
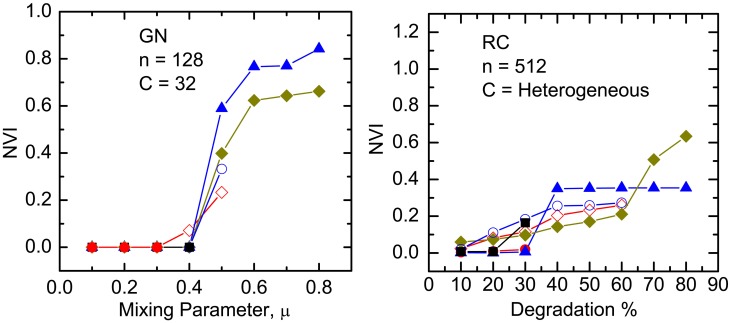
The *NVI* comparison on undirected and unweighted GN and RC benchmark networks. The size of community for RC ranges from 2 to 157. The legend is the same as in Figs 4 and 7.

The results of our *NVI* and *NMI* comparisons between the algorithms, on the various benchmark networks, are depicted in Figs 4 to 9. It must be noted that CLPA-GNR, LPA, GANXiS, NIBLPA and Infomap tend to detect a single community that consists of all the nodes in a network when *μ* or *D* is larger than certain values. Whenever an algorithm meets this condition, the detection is trivial and the *NVI* or *NMI* of the algorithm at that particular *μ* or *D* is not plotted in the graphs. Table 5 summarizes the values of *μ* and *D* for which the algorithms will obtain trivial detection in a network.

**Table 5:** The values of *μ* and *D* when CLPA-GNR, LPA, Infomap, GANXiS and NIBLPA obtain trivial detection.

Network	CLPA-GNR	LPA	Infomap	GANXiS	NIBLPA
S-LFR-SK-SC	*μ* ≥ 0.7	*μ* ≥ 0.7	N/A	*μ* ≥ 0.8	*μ* ≥ 0.7
S-LFR-SK-LC	*μ* ≥ 0.5	*μ* ≥ 0.6	N/A	*μ* ≥ 0.6	-
S-LFR-LK-SC	*μ* ≥ 0.7	*μ* ≥ 0.7	*μ* ≥ 0.7	*μ* ≥ 0.7	*μ* ≥ 0.7
S-LRF-LK-LC	*μ* ≥ 0.6	*μ* ≥ 0.6	*μ* ≥ 0.6	*μ* ≥ 0.7	*μ* ≥ 0.6
B-LFR-SK-SC	*μ* ≥ 0.8	*μ* ≥ 0.7	N/A	-	-
B-LFR-SK-LC	*μ* ≥ 0.6	*μ* ≥ 0.7	N/A	*μ* ≥ 0.8	-
B-LFR-LK-SC	*μ* ≥ 0.8	*μ* ≥ 0.8	-	N/A	N/A
B-LFR-LK-LC	*μ* ≥ 0.7	*μ* ≥ 0.7	-	N/A	N/A
GN	*μ* ≥ 0.5	*μ* ≥ 0.6	*μ* ≥ 0.4	*μ* ≥ 0.6	N/A
RC	*D* ≥ 40%	*D* ≥ 70%	*D* ≥ 40%	*D* ≥ 70%	N/A

A dash symbol shows that a method does not obtain any trivial solution up to *μ* = 0.8 in a particular benchmark network. *N*/*A* is shown when a method was not performed in a particular network.

Figs 4 to 6 depict the results of a comparison between the CLPA-GNR with the other LPA based algorithms. Overall, CLPA-GNR outperforms the other LPA based algorithms in all the LFR benchmark networks. The CLPA-GNR shows outstanding performance in S-LFR-LK-SC and S-LFR-LK-LC cases (Fig 5). It obtains perfect detection (*NMI* = 1) at *μ* = 0.6 and *μ* = 0.5 in S-LFR-LK-SC and S-LFR-LK-LC, when the *NMI* of the NIBLPA and GRANXiS are below 0.95. The only disadvantage of the CLPA-GNR is the trivial detection, where it obtains trivial detection at lower *μ* values.

At first glance, the result of the LPA is comparable to that for the CLPA-GNR when it does not obtain trivial detection. However, it must be noted that the detection of LPA is non-deterministic. In this study, the LPA is run for 1000 times and the results with highest modularity are reported. Although the LPA can detect communities in linear computation time, the large number of runs that are required, to get the best available results, causes it to lose its computation time advantage. Furthermore, it is not guaranteed that the LPA can always obtain the best results in a fixed amount of runs.

The results for the CLPA-GNR algorithm is compared to the results from the other algorithms (Infomap, RN and Blondel) in Figs 7, 8 and 9. It must be noted that GANXiS is included in the GN and RC cases (Fig 9). Generally, the results depicted in these figures suggest that the CLPA-GNR performs equally well as Infomap and RN in the LFR benchmark networks as long as it does not get any trivial detection in the networks. CLPA-GNR can always detect communities that match perfectly with the ground truth communities of the LFR benchmark networks, regardless of the size of the networks and communities. We also find that CLPA-GNR has a slightly better performance than Infomap in the GN benchmark networks, as it is able to detect communities that agree completely with the ground truth communities when *μ* ≥ 0.3 (Fig 9). On the other hand, CLPA-GNR is somewhat outperformed by Infomap in the B-LFR-LK-LC (Fig 8) and RC (Fig 9) cases. For example in B-LFR-LK-LC, CLPA-GNR obtains trivial detection while Infomap continues to give excellent detection even at *μ* = 0.7. Although CLPA-GNR is capable of obtaining non-trivial detection at 30% degradation in the RC benchmark network, this detection is not as good as that with the Infomap.

The Blondel algorithm, which focuses on optimizing the modularity, performs satisfactorily in all the benchmark networks. Even though it never gets a trivial detection, in any value of *μ* or *D*, it cannot detect communities as accurately as the other algorithms in all the benchmark networks. Similar to Blondel, RN can also always avoid trivial detection regardless of the values of *μ* or *D*. However, the detection of RN is generally better than that for Blondel.

It should be noted that the *NVI* or *NMI* increases or decreases drastically beyond certain values of *μ* or *D*. The sharp increment or decrement in the *NVI* or *NMI* indicates the ineffectiveness of an algorithm in coping with networks that have certain level of weak community structure. In spite of the fact that the detection may be non-trivial, the potentially large number of errors in the detected communities renders the results impractical for analysis purposes.

### Analysis on Real-World Networks

Table 6 depicts the *NVI* of 4 real-world networks with ground truth communities. CLPA-GNR performs very well by having the best detection performance in both the Karate and the Football networks. Although RN gives the best detection for the Pol-books network, the result of the CLPA-GNR is equivalent to that for RN.

**Table 6:** The *NVI* of real-world networks with ground truth communities. Bold values are the best detection algorithm for each network.

Network	CLPA-GNR	GANXiS	Infomap	RN	Blondel	LPA
Karate	**0.0639**	0.2212	0.1438	0.2289	0.2212	0.2212
Dolphin	0.2382	0.1957	0.2503	**0.0000**	0.2258	0.1708
Pol-books	0.2021	0.2055	0.2237	**0.2001**	0.2159	0.2055
Football	**0.0755**	0.1394	0.0902	0.0784	0.1148	0.1095

Table 7 shows the *Q* for all the real-world networks studied in this paper. It is not surprising to observe that the Blondel algorithm, which focuses on the optimization of the modularity, obtains the highest *Q* in more than half of the listed networks. Nonetheless, the CLPA-GNR is able to obtain results that are fairly close to the best *Q* values, except for the karate and jazz networks. The *Q*_*ds*_, which is perhaps a better alternative to *Q*, for the networks are depicted in Table 8. The performance of the CLPA-GNR is also acceptable under this measurement. Although there are distinct differences between the detections of the CLPA-GNR and the best algorithm results in some of the networks, the CLPA-GNR still exhibits satisfactory performance when compared to the other results. For instance, except for the best detections, it outperforms the other methods in the football and email networks. However, the detections of the CLPA-GNR do not agree with those from the other algorithms in the karate and jazz networks.

**Table 7:** The modularity (*Q*) for all the real-world networks. Bold values are the best detection algorithm for each network.

Network	CLPA-GNR	GANXiS	NIBLPA	Infomap	RN	Blondel	LPA
Karate	0.303	0.416	**0.423**	0.402	0.406	0.420	0.416
Dolphin	0.513	0.524	0.521	0.524	0.379	**0.527**	**0.527**
Pol-books	0.514	0.526	0.497	**0.527**	**0.527**	**0.527**	0.526
Football	0.601	0.604	0.582	0.603	0.601	0.604	**0.605**
Jazz	0.282	0.443	-	0.443	0.288	**0.445**	0.443
E. Coli	0.749	0.763	-	0.707	0.771	**0.777**	0.771
Email	0.520	0.548	0.427	0.536	0.008	**0.570**	0.558
Power	0.888	0.809	-	0.760	-	**0.934**	0.817
Collab.	0.796	-	-	**0.848**	-	0.844	0.781

**Table 8:** The modularity density (*Q*_*ds*_) for all the real-world networks. Bold values are the best detection algorithm for each network.

Network	CLPA-GNR	GANXiS	Infomap	RN	Blondel	LPA
Karate	0.182	0.234	0.217	**0.240**	0.230	0.234
Dolphin	0.187	0.189	**0.213**	0.136	0.187	0.187
Pol-books	0.183	0.192	**0.199**	0.190	0.191	0.192
Football	0.489	0.417	0.474	**0.491**	0.450	0.449
Jazz	0.187	0.215	**0.220**	0.205	0.213	0.215
E. Coli	0.116	0.153	0.087	**0.154**	0.116	0.142
Email	0.057	0.056	**0.088**	0.015	0.041	0.054
Power	0.055	0.144	0.003	-	0.019	**0.163**

In general, the performance of the CLPA-GNR is adequate under both of the *Q* and *Q*_*ds*_ metrics. Although LPA and GANXiS seem to have better detections than the CLPA-GNR in terms of *Q* and *Q*_*ds*_, it must be noted that both of them are non-deterministic algorithms. In order to obtain their results as shown in Tables 7 and 8 a total of 1000 runs are executed, and the best detections that show the highest possible *Q* or *Q*_*ds*_ values are then selected from those runs. As mentioned in the previous subsection, the non-deterministic nature of these methods hinders their performances in term of computational time. On the other hand, the CLPA-GNR is able to obtain deterministic detections that are comparable in quality to the detections of the LPA and GANXiS.

The CLPA-GNR obtains low *Q* and *Q*_*ds*_ values in the karate and jazz networks because it detects only 2 communities in those networks, while the other algorithms detect more than 2 communities. Despite having low *Q* and *Q*_*ds*_ for the karate network, the communities that are detected by the CLPA-GNR agrees very well with the ground truth communities of the network. In fact, as shown in Tables 6, 7 and 8, the highest *Q* or *Q*_*ds*_ value does not always indicate that the detected communities agree with the ground truth communities, which is a potentially serious limitation.

## Conclusions

In this paper, we addressed the non-deterministic nature of the LPA that is caused by its random label update sequences and random tie breaking process. Despite its superior computation time performance, the instability of the LPA reduces its robustness in detecting community. In order to tackle that problem, we proposed a modified LPA (the CLPA-GNR) with fixed label updated sequences. We also introduced a parameter to break a tie between communities. In addition, some steps are taken in the CLPA-GNR to refine the accuracy of detection, such as the update of labels in the CLPA-GNR is constrained by conditions which are gradually relaxed, as well as the recursive reallocation of grouped nodes throughout the CLPA-GNR. By implementing these modifications, we successfully improved the robustness and accuracy of the LPA. Aside from yielding deterministic results in networks of various sizes, the quality of community detection of the CLPA-GNR was proven to be satisfactory in this investigation. The CLPA-GNR performed extremely well in the LFR and GN benchmarks, while doing quite well in the RC benchmark. However, it suffers from trivial detection when the community structure of a network is weakened to a certain extent. Nonetheless, the same phenomenon can be observed in the original LPA as well as with Infomap, NIBLPA and GANXiS. The performance of the CLPA-GNR in real-world networks is notable too. Although the CLPA-GNR did not always obtain partition with the highest *Q* or *Q*_*ds*_, the fact that it gave good agreement between the detected communities, with the ground truth communities of some real-world networks, should not be overlooked.

There is still room for improvement in the performance of the CLPA-GNR, such as the extension of the CLPA-GNR into directed and weighted networks. As the LPA works in bipartite networks [39], it is expected that the CLPA-GNR can also be implemented into bipartite networks. The parallelization of the CLPA-GNR is a valid improvement too. Nevertheless, the trivial detection in certain networks remains the most important issue with it at this stage. The robustness of the CLPA-GNR will be improved greatly if that issue can be resolved in the future.
